# Cristaluria por sulfadiazina en paciente con nefropatía lúpica

**DOI:** 10.1515/almed-2022-0008

**Published:** 2022-06-15

**Authors:** María J. Ruiz Álvarez, Sergio Molina-Blas, Marta Barrionuevo-González, María E. Peñas-Lorite, José M. Gasalla-Herraiz

**Affiliations:** Servicio de Análisis Clínicos, Hospital Universitario Príncipe de Asturias, Alcala de Henares, Madrid, España

**Keywords:** cristaluria, sulfadiazina, síndrome nefrótico, cruz de Malta, lupus

## Abstract

**Objetivos:**

Se estima que el 29% de los pacientes tratados con sulfadiazina pueden desarrollar un fallo renal agudo. Para su diagnóstico debemos recurrir a un análisis del sedimento urinario.

**Caso clínico:**

Paciente de 71 años que consulta por pérdida de agudeza visual en el contexto de un brote de Lupus eritematoso sistémico (LES). Se diagnosticó de necrosis retiniana aguda, pendiente de filiación etiológica. Se inició empíricamente tratamiento con sulfadiazina. En los estudios analíticos de seguimiento se solicita sedimento urinario, en el cual se objetivan los siguientes hallazgos: pH 6, 30–50 hematíes/campo, células epiteliales de vías bajas y de transición, cilindros hialinos, gotas de grasa o cuerpos de cruz de Malta y abundantes cristales de sulfadiazina. Se comunica este hallazgo al Servicio de Nefrología y se suspende el tratamiento de forma inmediata.

**Conclusiones:**

La sulfadiazina es un antibiótico de la familia de las sulfamidas. Su cristalización en el interior de los túbulos renales puede causar nefritis instersticial aguda. Estos cristales adoptan una morfología muy diversa en función del metabolito que cristalice: las formas inalteradas precipitan en forma de cristales densos y globulares, o como en el caso de nuestro paciente, los cristales adoptan forma de espigas de trigo en abanico.

## Caso clínico

Presentamos el caso de una paciente de 71 años diagnosticada de lupus eritematoso sistémico (LES), que acudió al Servicio de Oftalmología por pérdida brusca de agudeza visual unilateral y fue ingresada con el diagnóstico de necrosis retiniana aguda.

En el contexto de su patología de base destacan como antecedentes una vasculitis retiniana con desprendimiento seroso de retina y una nefritis lúpica proliferativa difusa grado 4, siendo catalogada en el momento del ingreso de enfermedad renal crónica (ERC G3) con niveles de creatinina de 1,4 mg/dL, urea 70 mg/dL, filtrado glomerular (FG) estimado (CKD-EPI) de 42 mL/min/1,73 m^2^ y proteinuria en rango nefrótico de 28 g/24 h. Recibía tratamiento habitual con prednisona 60 mg/24 h en pauta descendente, micofenolato mofetilo 3 g/24 h y ciclos de ciclofosfamida 900 mg/3 semanas.

Al ingreso por necrosis retiniana, y a la espera del despistaje microbiológico, comenzó a ser tratada de forma empírica con trimetoprim-sulfametoxazol (TMT-SMX) 160 mg/800 mg cada 12 h por sospecha de toxoplasmosis ocular. La aparición de lesiones cutáneas en miembros inferiores compatibles con vasculitis leucocitoclástica mediada por TMT-SMX obligó a suspender el tratamiento y a sustituir por la pauta clásica para toxoplasmosis, pirimetamina 50 mg/24 h y sulfadiazina 1 g/6 h [1, 2].

**Figura 1:**
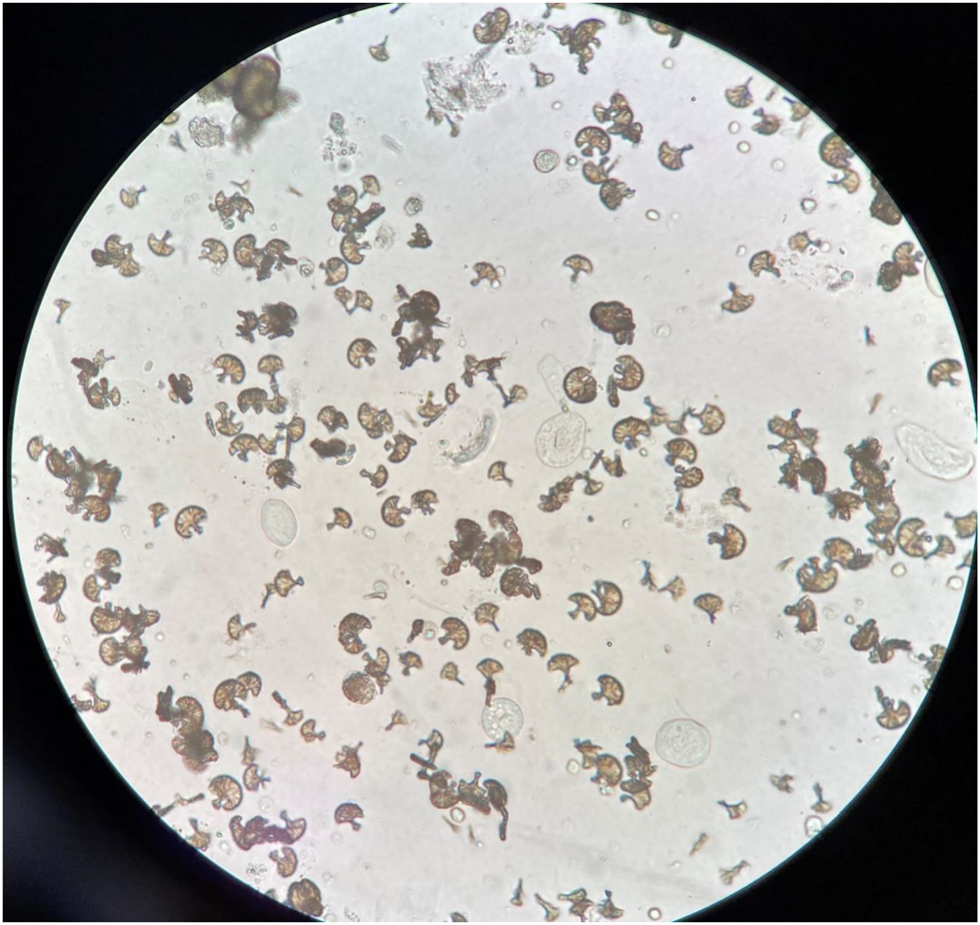
Cristales de sulfadiazina en sedimento urinario, 40×. (Fotografía realizada por los autores).

Tras la introducción de dicho tratamiento se objetivó un empeoramiento brusco de la función renal, con cifras de creatinina de 2,2 mg/dL con un FG estimado (CKD-EPI) de 21 mL/min/1,73 m^2^. En el estudio del sedimento urinario destacaban los siguientes hallazgos: pH 6, proteínas 300 mg/dL, 30–50 hematíes/campo, presencia de cilindros hialinos y de células epiteliales de vías bajas y presencia de abundantes cristales medicamentosos de sulfadiazina y cuerpos grasos ovales (Figuras 1 y 2). Ante este hallazgo, se alertó a Nefrología.

**Figura 2:**
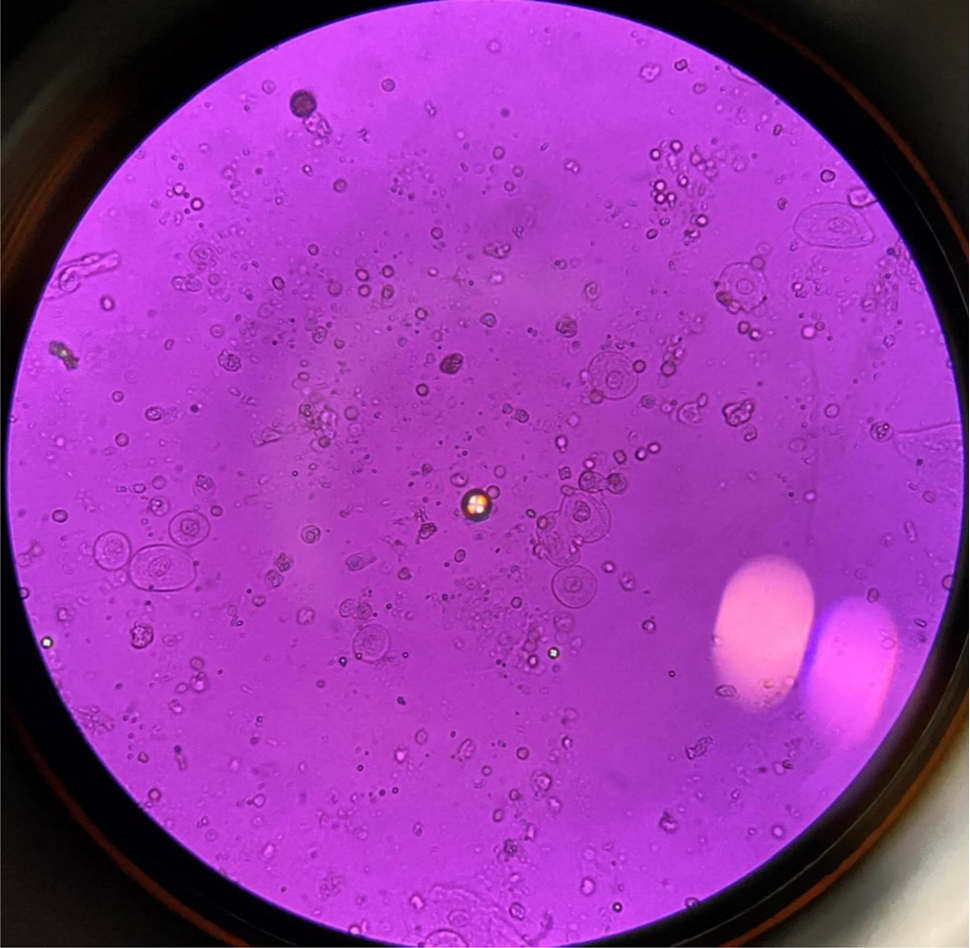
Cuerpos de cruz de Malta en orina de paciente con síndrome nefrótico, 40×. (Fotografía realizada por los autores).

## Discusión

Los cristales de sulfadiazina en gavilla de trigo con unión excéntrica proceden de la cristalización del metabolito hepático del fármaco, la acetilsulfadiazina [3, 4]. Existen distintos factores que favorecen la precipitación del fármaco, como son pH urinario ácido, hipoalbuminemia, situaciones que favorecen la depleción de volumen, concentración del fármaco en orina, dosis administrada y el tratamiento concomitante con fármacos que acidifiquen la orina [5, 6].

El tratamiento consiste en alcalinización de la orina e hidratación abundante para mantener una diuresis diaria superior a 2 L. Debe suspenderse el fármaco y valorar la reintroducción de forma individualizada cuando se corrija la función renal.

Los cuerpos grasos ovales son gotas de grasa de distinto tamaño, generalmente pequeño, que proceden de la ruptura de células epiteliales ricas en grasa. Cuando las gotas son de colesterol y se observan con microscopio de luz polarizada, adoptan una forma característica denominada cruz de Malta. Se pueden encontrar en distintas patologías tubulointersticiales, siendo bastante características del síndrome nefrótico.

En el caso de la paciente descrita, la complejidad de su patología de base supuso importantes problemas a la hora de adoptar medidas terapéuticas. No se consideró el pH urinario como factor precipitante de la cristalización del fármaco, sino más bien un conjunto de factores relacionados con su patología de base, como fueron el tratamiento con diuréticos, la hipoalbuminemia y la depleción de volumen.

Se suspendió sulfadiazina de forma inmediata y no volvió a reintroducirse por la mala evolución de su patología renal. Posteriormente, los estudios microbiológicos confirmaron que la etiología de la necrosis retiniana era herpética.

La suspensión de diuréticos y la repleción de volumen se hizo de forma muy gradual también por la mala evolución de su patología renal.
